# Linking clinical presentation and histopathological data in perforation peritonitis

**DOI:** 10.6026/973206300222097

**Published:** 2026-04-30

**Authors:** Kusum Mahour, Pradeep Rathore, Aasha Baghel

**Affiliations:** 1 Department of General Surgery, Government Medical College, Datia, Madhya Pradesh, India; 2 Department of Pathology, Government Medical College, Datia, Madhya Pradesh, India

**Keywords:** Perforation peritonitis, histopathology, clinical correlation, laparotomy, gastrointestinal perforation

## Abstract

Perforated peritonitis presents a diagnostic challenge because clinical manifestations often poorly correlate with the underlying histopathological severity and etiology. This prospective observational study included 50 patients with perforated peritonitis who underwent emergency laparotomy, assessing clinical parameters such as presenting symptoms, duration of illness, pain location, hemodynamic status and laboratory findings. Intraoperative details including the site and size of perforation, degree of peritoneal contamination and associated pathology were recorded and histopathological examination was performed on resected specimens and biopsy samples from perforation margins. Statistical analysis revealed a strong association between clinical presentation and histopathological severity, confirming that clinical features can reflect the underlying pathological burden. Thus, histopathological analysis is indispensable for accurate etiological diagnosis and for guiding postoperative management in perforated peritonitis.

## Background:

Perforation peritonitis is a significant surgical emergency, defined as contamination of the peritoneal cavity resulting from loss of gastrointestinal tract integrity. It is a major cause of emergency surgical hospitalizations. It is linked to a high morbidity and mortality rate, especially in low- and middle-income nations where possible delay in presentation is an issue [1]. The etiology of perforated peritonitis varies geographically. Lower gastrointestinal tract perforations [especially after malignancy and diverticular disease] are most common in Western countries. Still, upper gastrointestinal perforations [especially after peptic ulcer disease] are the most common in developing countries [2]. Typhoid fever and TB are other infectious agents associated with ileal perforations in the tropics [3]. The patients will normally show up clinically with a sudden onset, severe abdominal pain, vomiting, abdominal distension, fever and the signs of peritoneal irritation, including guarding and rigidity. Nevertheless, the presentation can vary depending on the perforation location, pre-treatment time, immune status and pathology [4]. Early clinical identification of disease severity must be done since late intervention leads to the development of septic shock, organ failure and fatality [5]. Radiological tests, especially erect abdominal or chest radiographs that show pneumoperitoneum, are helpful in diagnosis, although they do not give any information on the underlying pathology that causes the perforation [6]. Therefore, certain diagnoses are frequently based on findings during the operation and on histologic analysis of tissue taken at the perforation margins. Histopathology is critical for determining the specific cause of perforation, distinguishing benign from malignant disease, identifying specific infections such as tuberculosis or typhoid and assessing the extent of inflammation, necrosis, or malignancy [7]. The information plays a vital role in guiding post-operative treatment, including the choice of antibiotics, the need for oncology treatment and long-term follow-up. Several studies have shown that the pathological process and disease duration tend to be reflected in the clinical severity of presentation. Late-presenting patients often show progressive parenchymal inflammation, tissue necrosis, or malignancy on histopathology, which negatively influences prognosis [8]. Therefore, it is of interest to assess the correlation between clinical presentation and histopathological results in patients with perforated peritonitis, to improve diagnostic accuracy and management and to predict patient outcomes.

## Materials and Methodology:

## Study design:

A prospective observational hospital-based study

## Study setting:

The study was conducted in the Department of General Surgery at a tertiary-care teaching hospital over 6 months.

## Study population:

All patients present with clinical and radiological diagnosis of perforated peritonitis that underwent emergency laparotomy during the study period.

## Sample size:

A total of 50 patients fulfilling the inclusion criteria were enrolled consecutively.

## Inclusion criteria:

[1] Patients aged ≥18 years

[2] Patients with clinical features suggestive of perforation peritonitis

[3] Radiological evidence of pneumoperitoneum or free fluid

[4] Patients undergoing emergency laparotomy

[5] Patients providing informed consent

## Exclusion criteria:

[1] Primary peritonitis without perforation

[2] Traumatic perforations requiring damage control surgery only

[3] Patients unfit for surgery

[4] Refusal to participate

[5] Previously operated perforation cases referred after surgery

## Data collection:

## Clinical assessment:

A detailed clinical history was obtained, including:

[1] Duration of abdominal pain

[2] Vomiting, fever, abdominal distension

[3] History of peptic ulcer disease, drug intake (NSAIDs/steroids)

[4] History of enteric fever or tuberculosis

[5] Comorbidities

## Physical examination included:

[1] Pulse, blood pressure, temperature

[2] Signs of dehydration or shock

[3] Abdominal tenderness, guarding, rigidity

[4] Bowel sounds

[5] Rectal examination findings

## Laboratory investigations:

[1] Complete blood count

[2] Serum electrolytes

[3] Renal function tests

[4] Liver function tests

[5] Blood culture (when indicated)

## Radiological evaluation:

[1] Erect abdominal or chest X-ray for pneumoperitoneum

[2] Ultrasonography of the abdomen for free fluid/collection

[3] CT abdomen (in selected stable patients)

## Intraoperative findings:

During laparotomy, the following were recorded:

[1] Site of perforation

[2] Size of perforation

[3] Degree of peritoneal contamination

[4] Presence of pus/fecal matter

[5] Underlying pathology suspected intraoperatively

[6] Surgical procedure performed

## Histopathological examination:

[1] Biopsy samples were taken from the edges of the perforation or the resected specimen

[2] Samples were fixed in 10% formalin

[3] Processed and stained with Hematoxylin & Eosin

[4] Special stains used when required (e.g., Ziehl-Neelsen for tuberculosis)

## Histopathology was evaluated for:

[1] Acute/chronic inflammation

[2] Necrosis or gangrene

[3] Granuloma formation

[4] Malignancy

[5] Specific infection

## Outcome measures:

## Primary outcome:

Correlation between clinical severity and histopathological diagnosis

## Secondary outcomes:

[1] Relationship between duration of symptoms and pathological severity

[2] Association between the site of perforation and the underlying etiology

[3] Postoperative complications and mortality

## Statistical analysis:

[1] Data entered into MS Excel and analyzed using SPSS software

[2] Continuous variables expressed as mean ± SD

[3] Categorical variables expressed as percentages

[4] Chi-square test used for correlation between clinical and histopathological variables

[5] p-value <0.05 considered statistically significant

## Results:

A total of 50 patients with perforated peritonitis were included in the study. The age ranged from 18 to 75 years, with a male predominance. Most patients presented with acute abdominal pain, vomiting and signs of generalized peritonitis. Table 1 (see PDF) shows the distribution of presenting clinical features as percentages. Acute abdominal pain was universal. Guarding and rigidity were present in more than 92% of patients, indicating widespread peritoneal inflammation and about one-fourth of patients presented with shock, suggesting late presentation and severe sepsis. Table 2 (see PDF) demonstrates the distribution of perforation sites. Duodenal perforation accounted for nearly half of the cases, reflecting the high prevalence of peptic ulcer disease. Ileal perforations were the second most common, typically related to infectious etiologies. Gastric and colonic perforations were less frequent. Peptic ulcer disease was the most common cause, accounting for more than half of the patients. Infectious causes accounted for over one-fourth of cases, underscoring the importance of regional epidemiology. Malignancy-related perforation was relatively rare but clinically significant (Table 3 - see PDF). Table 4 (see PDF) demonstrates a strong correlation between delayed presentation and severity of histopathological findings. Patients presenting after 24 hours showed a markedly higher incidence of necrosis and severe inflammation, indicating worse prognosis and increased post-operative complications.

## Discussion:

One of the most commonly met surgical emergencies in developing countries and a leading cause of emergency laparotomy work is perforated peritonitis. The clinical presentation and histopathological findings correlate strongly, as shown in the present study. The presence of abdominal pain was found in 100 percent of patients in the current study, with 92 percent reporting guarding and rigidity, which shows generalized peritonitis. Singh et al. reported similar results, with peritoneal signification present in 90% of cases [9]. Abdominal pain was also reported in almost all cases of patients with perforated peritonitis [10]. These results support the fact that acute abdominal pain in the presence of peritoneal signatures and indicators is characteristic of gastrointestinal perforation. This current study revealed duodenal perforation in 44 percent of patients and ileal perforation in 28 percent. This trend is similar to that reported by Jhobta *et al.* [1], who found a 45160% incidence of gastroduodenal perforations in India [3]. Western literature, on the other hand, records a greater case of lower gastrointestinal perforation that can be attributed to malignancy or diverticular disease [4]. The fact that the majority of upper gastrointestinal perforations in this study are due to regional epidemiological differences. In the current study, histopathological analysis found that 52, 28 and 12 percent of patients had peptic ulcer disease, infectious and appendicular perforation, respectively and 8 percent had malignancy. Such results can be compared with those of Chakma *et al.* who found peptic ulcer disease as the primary cause in 5055% of patients [11]. Typhoid, tuberculosis and infectious perforations, especially typhoid, are still prevalent in developing nations and were also present in 25-30% of cases during one study done by Yadav *et al.* [6]. One notable finding in the current research study was the correlation between delayed presentation and the extent of histopathological alterations. In patients presenting late (after 24 hours), there was severe inflammation or necrosis in almost 72% of cases compared to only 30% in the early presenters. Correlations between treatment delay and severity of the pathology were similar and those with thy showed significantly higher complication rates in late presenters [12, 13-14]. There is an implication of the significance of diagnosis and timely surgical intervention. In the current study, malignancy was among the 8 percent of perforations, which is comparable to the 5-10 percent in the past Indian research. Malignant perforations are, however, less frequent, have a poorer prognosis and necessitate further oncological assessments [15, 16-17]. Overall, the results of this study confirm that histopathological diagnosis is closely related to clinical severity, symptom duration and perforation location. This can be used to predict underlying pathology and make surgical decisions through early clinical recognition.

## Conclusion:

We report a strong correlation between clinical manifestations and histopathological findings in perforated peritonitis. The most reliable clinical indicator is acute abdominal pain that is accompanied by peritoneal signs. The most frequent underlying pathology remains peptic ulcer disease and then there are infectious causes. The histopathological study of the margins of perforation is necessary to confirm the etiology, malignancy, or infection and to inform post-operative treatment.
